# Stem cell status and prognostic applications of cuproptosis-associated lncRNAs in acute myeloid leukemia

**DOI:** 10.3389/fcell.2024.1549294

**Published:** 2025-01-14

**Authors:** Zhuodong Chai, Zhongyue Yuan, Yifei Chen

**Affiliations:** ^1^ Department of Pharmaceutical Sciences, Irma Lerma Rangel School of Pharmacy, Texas A&M University, College Station, TX, United States; ^2^ Department of Industrial and Molecular Pharmaceutics, Purdue University, West Lafayette, IN, United States; ^3^ Department of Hematology, Jiangdu People’s Hospital, Yangzhou, China

**Keywords:** cuproptosis, lncRNA, AML prognostic model, stem cells, drug sensitivity, immune microenvironment

## Abstract

**Introduction:**

Acute myeloid leukemia (AML), a highly heterogeneous hematological malignancy, remains a major challenge in adult oncology. Stem cell research has highlighted the crucial role of long noncoding RNA (lncRNA) in regulating cellular differentiation and self-renewal processes, which are pivotal in AML pathogenesis and therapy resistance.

**Methods:**

This study explores the relationship between cuproptosis-related lncRNAs and AML prognosis, providing novel insights into their impact on hematopoietic stem and progenitor cells.

**Results:**

We collected clinical information from 214 AML patients in our center and analyzed the association between granulocyte recovery after chemotherapy, cuproptosis, and prognosis. Additionally, we developed a prognostic model—the cuproptosis-associated long noncoding RNA prognostic model (CRLPM)—y analyzing data from The Cancer Genome Atlas (TCGA). Patients were stratified into high- and low-risk groups based on CRLPM, revealing significant survival differences. High-risk patients demonstrated lower sensitivity to chemotherapeutic agents such as Axitinib, GSK429286A, Navitoclax, and ZM-447439, underscoring the need for alternative therapeutic strategies.

**Discussion:**

CRLPM offers a promising framework for integrating stem cell-focused approaches into personalized treatment regimens, paving the way for precision medicine in AML management.

## Introduction

Acute myeloid leukemia (AML), the most common form of acute leukemia in adults, is a highly aggressive and heterogeneous hematologic malignancy, with increased incidence with age. In 2024, there are 20,800 estimated new cases in the United States (Siegel et al., 2024). AML is characterized by the rapid proliferation of immature myeloid cells, which accumulate in the bone marrow and peripheral blood (Pimenta et al., 2021), leading to impaired normal hematopoiesis and causing symptoms like anemia, thrombocytopenia, and infections (Albrecht, 2014). AML exhibits significant genetic and molecular heterogeneity, with a complex range of gene mutations and chromosomal abnormalities, including key alterations in genes such as FLT3, NPM1, DNMT3A, and TP53 in AML patients (Ali and Salih, 2023). These complexities not only enhance the disease’s aggressiveness but also pose significant treatment challenges (Abaza et al., 2024). Moreover, AML originates from hematopoietic stem and progenitor cells (HSPCs), where dysregulated self-renewal and differentiation processes contribute to leukemogenesis. This stem cell involvement underscores the importance of targeting these cells in therapeutic strategies. The genetic and epigenetic heterogeneity of AML results in wide variations in treatment responses and survival outcomes and therefore understanding the molecular mechanisms underlying AML pathogenesis is critical to improving diagnosis and treatment. Long-term survival for AML remains low, especially for elderly and high-risk patients, despite the effectiveness of traditional therapies (Chen et al., 2023). Recent advances in precision medicine and targeted therapies have shown promise in improving outcomes for certain patient subgroups. However, drug resistance and relapse continue to be critical issues that need to be addressed (Watts and Nimer, 2018).

Noncoding RNA, especially long noncoding RNA (lncRNA), plays a key role in cancer progression and treatment responses by regulating gene expression (Statello et al., 2020; Mattick et al., 2023). lncRNA is also involved in key biological processes that drive the pathogenesis of AML, such as chromatin remodeling, transcriptional regulation, and signaling pathway regulation (Gourvest et al., 2019; Mer et al., 2018). Studies have shown that dysregulated lncRNAs affect the proliferation, differentiation, and survival of leukemia cells, leading to disease progression and treatment resistance (Gao et al., 2020; Izadirad et al., 2021). Regulating specific lncRNAs may alter the expression of genes that regulate leukemogenesis, thereby providing new strategies for more effective and targeted AML treatments (Mishra et al., 2022). Although the general role of lncRNAs in AML is well-studied, a systematic analysis of lncRNAs specifically associated with cuproptosis has not been conducted. Such an analysis could uncover lncRNAs that modulate the response of AML cells to copper-induced stress, offering new insights into both prognosis and potential treatment avenues (Xie et al., 2023; Springer et al., 2024). Cuproptosis is a regulated cell death pathway induced by intracellular copper accumulation, providing a potential mechanism for anti-cancer treatment based on manipulating copper levels within tumor cells (Xie et al., 2023; Tsvetkov et al., 2022). It involves direct binding of copper to lipid acylases of the tricarboxylic acid (TCA) cycle, leading to protein aggregation and lethal protein stress (Tsvetkov et al., 2022). This interaction disrupts protein folding, causes aggregation, and leads to proteotoxic stress, which can ultimately kill the cell. This pathway is closely tied to mitochondrial function and depends on active TCA cycling and oxidative phosphorylation (Springer et al., 2024). This mechanism provides a unique approach to exploit the metabolic vulnerability of cancer cells, especially in AML, where altered metabolism supports the growth of leukemic cells (Wang X. et al., 2024; Wu et al., 2024). For cancer treatment, dysregulated copper metabolism is frequently observed in cancer cells, Aggressive tumors, in particular, often require higher copper levels to sustain rapid proliferation, making them more susceptible to copper-induced toxicity. In AML, for example, copper may activate key signaling pathways like PI3K-AKT and MAPK, which promote tumor growth and migration. This dependency on copper suggests that modulating intracellular copper levels to induce cuproptosis could be a promising therapeutic approach for cancers characterized by abnormal copper metabolism (Xie et al., 2023).

Recently, lncRNA-based models have been proposed as potential tools to improve AML prognosis by integrating molecular data beyond traditional gene mutations (Gourvest et al., 2019; Pan et al., 2017; Tian et al., 2019; Zhu et al., 2023; Zhao et al., 2021). However, cuproptosis-associated lncRNA impact prognosis in patients with AML has yet to be explored. This study investigated the prognostic model of cuproptosis-related lncRNAs in AML by detailed genomic and transcriptomic analyses. This study aims to develop a prognostic model based on cuproptosis-associated lncRNAs to improve AML patient stratification. To achieve this, we will utilize RNA sequencing data from the TCGA database and identify lncRNAs significantly correlated with overall survival using univariate Cox regression. Then, LASSO regression will optimize variable selection to ensure model robustness. Validation will be performed using Kaplan-Meier survival curves, ROC curves, and C-index calculations. Additionally, external data sets will help confirm CRLPM’s consistency and reliability across diverse patient populations. This model has the promising potential to provide personalized treatment strategy guidance for patients with different risk levels and could also provide guidance for medication recommendations to enhance therapeutic treatment. For high-risk patients, the model could identify individuals with low sensitivity to certain chemotherapies, thereby reducing unnecessary toxicity. Furthermore, CRLPM could support the identification of patients who may benefit from treatments targeting copper metabolism pathways, enhancing therapeutic outcomes in cases where conventional chemotherapy might be less effective.

## Methods and materials

### Patients

A total of 214 patients with newly diagnosed and receiving treatment for non-M3 AML at the Yangzhou Jiangdu People’s Hospital between January 2018 and August 2022 were enrolled. Risk stratification of patients was classified according to the 2017 ELN risk criteria. Informed consent was obtained from all patients before data collection. The study was approved by the Research Ethics Review Committee of Yangzhou Jiangdu People’s Hospital and was conducted following the Declaration of Helsinki.

### Transcriptomic data, mutation profiles and clinical information of TCGA

**FIGURE 1:**
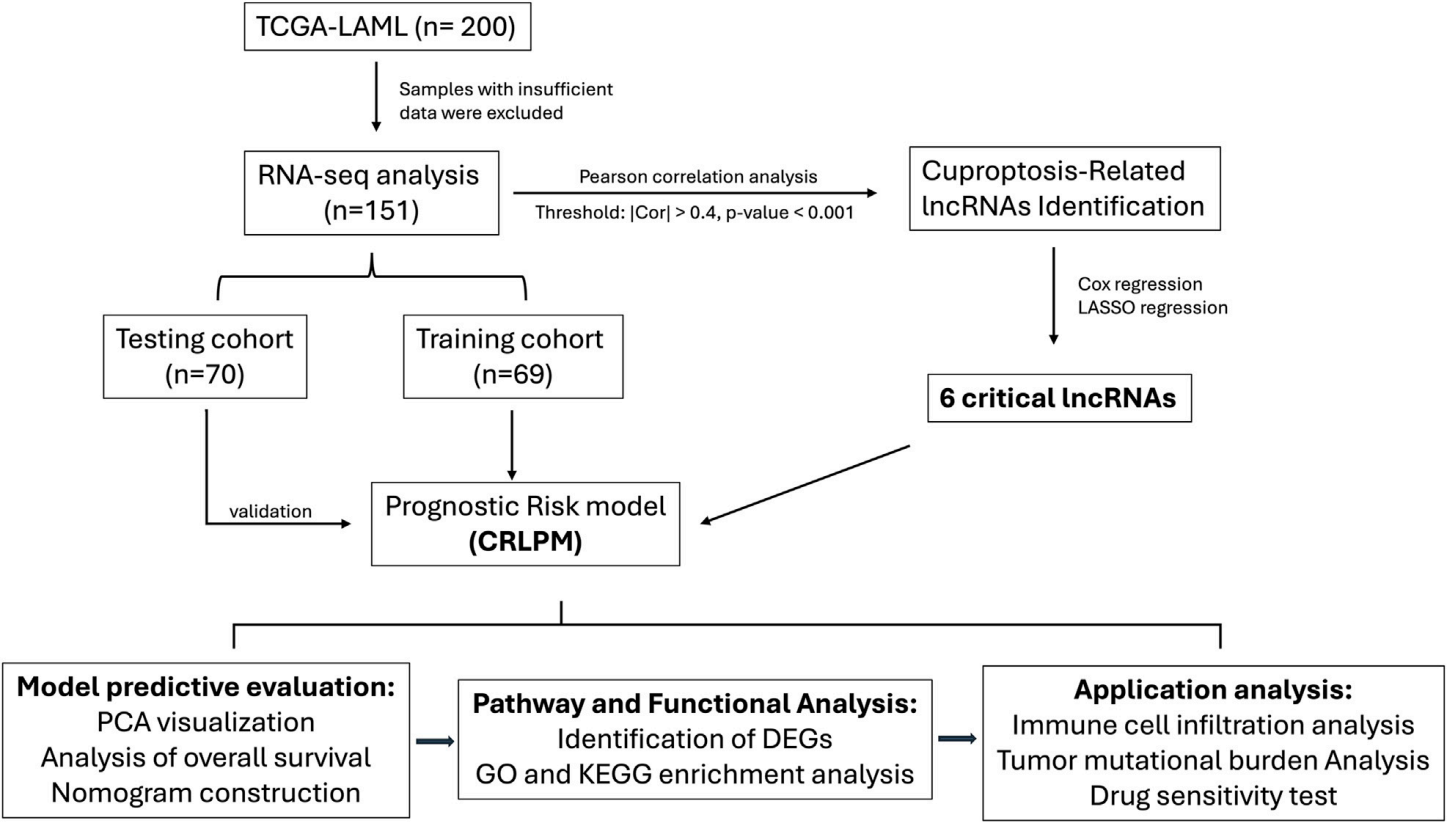
Flow chart of the study design.

The dataset of RNA sequencing for this analysis includes a total of 200 acute myeloid leukemia (AML) samples, sourced from the Cancer Genome Atlas (TCGA) database as of December 2023 (https://portal.gdc.cancer.gov/). This dataset integrates extensive molecular and clinical data, facilitating a detailed examination of the relationship between long non-coding RNA (lncRNA) expressions and patient outcomes. The patient dataset spans an age range of 30–85 years, with a median age of 60 years old, and features an approximately equal distribution of male and female patients, thus reducing gender bias in the study. The data covers various histological AML subtypes, allowing for the investigation of subtype-specific lncRNA profiles (Figure 1).

Before conducting further analyses, RNA sequencing data were preprocessed to ensure data quality and consistency. Selection criteria included samples with complete clinical and genomic data and a sequencing depth of at least 30x. Samples with low quality or a missing rate exceeding 10% were excluded. To ensure comparability between samples, data were standardized and normalized prior to analysis. Normalization accounted for sequencing depth and gene length, reducing variability and batch effects.

The overall survival (OS) was applied to assess the prognostic value of lncRNAs. RNA sequencing data were analyzed using established bioinformatics tools; utilizing the STAR aligner, reads were mapped to the GRCh38 reference genome, and lncRNA expression was quantified by HTSeq-count, normalized for both gene length and sequencing depth. Differential expression analysis utilized DESeq2 to highlight lncRNAs linked to clinical outcomes. LncRNA annotations were provided by GENCODE database (https://www.gencodegenes.org/), and a subset of genes linked to cuproptosis was identified based on the research by Tsvetkov et al. (2022).

### Identification and analysis of cuproptosis-related long non-coding RNAs

To identify the association of lncRNAs and cuproptosis, we utilized the “limma” package in R to conduct a correlation analysis. We calculated the Pearson correlation coefficients between lncRNAs and cuproptosis-related genes. LncRNAs that demonstrated a correlation coefficient |Cor| > 0.4 and a p-value less than 0.001 were deemed significantly associated with cuproptosis. These threshold values were selected based on commonly accepted practices in similar transcriptomic studies within various subjects, which aim to balance the sensitivity and specificity of identifying meaningful correlations while minimizing false positives (Yin et al., 2021; Shi et al., 2017; Yao et al., 2024; Li et al., 2022; Zhang et al., 2024). We illustrated the interactions between these genes and the identified lncRNAs using Sankey diagrams, which were created employing the “ggplot2”, “ggalluvial”, and “dplyr” packages in R.

### Construction of a prognostic risk model

We partitioned the dataset into training and testing cohorts using a random assignment approach, facilitated by the ‘caret’ package in R. To ensure that clinical variables such as age, gender, and other relevant factors were evenly distributed between the two groups, stratified random sampling was employed. This method maintained the balance of important clinical characteristics across the training and testing sets, reducing potential biases and ensuring a more reliable validation of the model. We constructed cuproptosis-related lncRNA signatures using the training set and validated these signatures using the test set and the entire dataset. Univariate Cox proportional hazards regression analysis was performed on the cuproptosis-associated lncRNAs using the ‘survival’ package to identify those significantly linked to OS. To enhance the model’s accuracy and prevent overfitting, we employed the Lasso Cox regression via the ‘glmnet’ package in R. The optimal penalty parameter (λ) was determined through 10-fold cross-validation, selecting the value that minimized the Akaike Information Criterion (AIC) and Bayesian Information Criterion (BIC), both of which balance model complexity and goodness-of-fit. This approach ensures that the final model retains only the most relevant features while controlling for overfitting. Subsequently, we constructed a Cuproptosis-Related Long Non-Coding RNA Prognostic Model (CRLPM) based on multivariate Cox regression analysis. The risk score for each patient was calculated using the formula:

$$Risk\;score=\Sigma \;i=\ln\;Coef\left(i\right)\times Ex\left(i\right)$$

where Coef(i) is the regression coefficient of the multiple Cox regression analysis for each lncRNA, and Ex(i) is the normalized expression level of that lncRNA.

### Model evaluation and nomogram construction

To assess the effectiveness of prognostic model, patients in both the training and testing cohorts were categorized into high- and low-risk groups using the median risk score calculated from the training set. Kaplan-Meier survival curves were then generated to compare the survival outcomes between these groups. The prognostic precision of the model was further evaluated using receiver operating characteristic (ROC) curve analysis. The area under the curve (AUC) was determined employing the “survivalROC” and “timeROC” packages in R. The performance of the model was further validated by calculating the concordance index (C-index) with the “rms”, “survival”, and “pec” packages in R. Both metrics were used to assess the predictive accuracy and robustness of the model.

To further validate the robustness of the model, we employed four external datasets obtained from the Gene Expression Omnibus (GEO) database (https://www.ncbi.nlm.nih.gov/geo/). The following datasets were used: GSE12417 (242 adult patients with untreated AML), GSE37642 (562 samples from adult patients with AML), GSE71014 (104 *de novo* AML patients with normal karyotype), and GSE76009 (534 samples from adult patients with AML). All these datasets were based on array analysis, using tissues from bone marrow or peripheral blood mononuclear cells (GSE12417, GSE37642), bone marrow mononuclear cells (GSE71014), and human acute myeloid leukemia cells (GSE76009).

To ascertain the prognostic relevance of risk model, both multivariate Cox regression and univariate analyses were conducted to determine the model’s independent prognostic value. To assess the combined impact of risk scores and clinical-pathological factors on OS at 1, 3, and 5 years for AML patients, a nomogram was subsequently constructed by using the “rms”, “survival”, and “regplot” packages in RStudio, for visually depicting the predictive outcomes derived from the Cox regression analyses. Calibration curves, evaluated using the Hosmer-Lemeshow test, were used to determine the accuracy of the constructed nomogram models. This nomogram offers a graphical representation for estimating patient survival outcomes in regard to risk models and other factors in clinic (Iasonos et al., 2008).

Additionally, principal component analysis (PCA) was conducted to visualize the distribution of patients in high- and low-risk categories, using the “scatterplot3D” package.

### Pathway and functional analysis

RNA-seq reads were aligned using STAR software (v2.7.9a) with all default parameters. For differential expression gene (DEG) analysis, the DESeq2 package (v1.30.1) was used, with thresholds set at p-value <0.001 and |fold change| > 1. All p-values from statistical tests were adjusted for multiple testing using the Benjamini-Hochberg method to reduce false-positive rates. Following the identification of DEGs, we conducted a series of functional enrichment analyses to understand relevance of these genes in biologic.

Gene Ontology (GO) enrichment analysis was conducted to identify significantly overrepresented GO terms in the categories of biological process (BP), cellular component (CC), and molecular function (MF), providing insight into the broader biological roles of the DEGs. Kyoto Encyclopedia of Genes and Genomes (KEGG) pathway analysis was also carried out to uncover key signaling pathways associated with the risk groups. For these analyses, we utilized several R packages, including “clusterProfiler” for the enrichment analysis, “org.Hs.eg.db” for human gene annotation, and “enrichplot” for visualizing the results.

### Analysis of tumor-infiltrating immune cells and immunotherapy response

Single-sample gene set enrichment analysis (ssGSEA) in R was applied to investigate the connection between the Cuproptosis-Related Long Noncoding RNA Prognostic Model (CRLPM) risk score and immune cell infiltration. This approach benefited to assess the levels of infiltration and the functional activities of tumor-infiltrating immune cells.

We employed single-sample gene set enrichment analysis (ssGSEA) in R to investigate the relationship between the Cuproptosis-Related Long Noncoding RNA Prognostic Model (CRLPM) risk score and immune cell infiltration. This enabled us to determine both the infiltration levels and functional activities of cacner-infiltrating immune cells. The outcomes of this analysis were depicted through a heatmap. Furthermore, to estimate the potential response to immunotherapy, we employed the Tumor Immune Dysfunction and Exclusion (TIDE) algorithm (Jiang et al., 2018), which is available online at http://tide.dfci.harvard.edu. The TIDE algorithm simulated tumor immune evasion mechanisms, offering insights into potential immunotherapy responses in the high- and low-risk patient groups.

### Calculation of tumor mutation burden (TMB) in AML

In this study, we quantified the TMB in AML samples to explore its potential role in disease progression and patient outcomes. Using the R package “maftools”, we analyzed mutation data and computed TMB scores for each patient (Mayakonda et al., 2018). The association between TMB and risk scores was assessed by dividing patients into high-risk and low-risk groups based on the CRLPM score. The mutation landscape and TMB distribution were visualized using waterfall plots, and survival analysis was developed to evaluate the influence of TMB and risk scores on OS.

### Drug sensitivity assessment

To develop the clinical application of the CRLPM model to treat AML, we calculated the half-maximal inhibitory concentration (IC50) of several chemotherapeutic drugs using the R package “pRRophetic” and its dependencies, including “car”, “ridge”, “preprocessCore”, “genefilter”, and “sva”. The Wilcoxon signed-rank test was employed to compare IC50 values between high-risk and low-risk groups for each drug. Visualization of the results was achieved using boxplots generated by the R package “ggplot2”. Kaplan-Meier curves were produced to illustrate cumulative survival probabilities, and the log-rank test was used to assess differences in survival rates between groups. Univariate and multivariate Cox proportional hazards models adjusted for survival-related covariates and estimated OS. The importance of prognostic factors was visually represented in forest plots.

### Statistical analysis

To handle missing data (≤5.0%), the random forest method was applied using the “mice” package in RStudio (version 4.3.2). For continuous variables, their distribution determined how they were presented: either as median values with interquartile ranges, or as means with standard deviations. The Wilcoxon rank-sum test was applied to evaluate differences between two groups for continuous data. Categorical data were summarized as proportions and analyzed using the chi-square test for comparisons between groups. Group differences were analyzed using one-way ANOVA for data following a normal distribution or the Kruskal-Wallis test for non-normally distributed data. Constrained cubic spline functions were utilized using the “rms” package to investigate potential non-linear relationships. Clustering results were visualized through heatmaps generated by the “Pheatmap” package. All statistical analyses were carried out by using packages such as, “ggplot2”, “rms” “PredictABLE”, “risk regression”, and “survminer” in RStudio (version 4.3.2).

## Results

### Impact of neutropenia duration and copper metabolism on post-chemotherapy hematopoietic recovery in AML patients

Among the 214 AML patients analyzed, the duration of neutropenia (defined as granulocyte recovery time) showed a significant correlation with hematopoietic stem cell and white blood cell recovery rates. Patients with neutropenia duration exceeding 25 days exhibited delayed recovery of stem cells and granulocytes, with a median age of 38 years (IQR: 29–46), compared to 35 years (IQR: 25–43.5) in those with shorter neutropenia durations. Additionally, the cohort with prolonged neutropenia had higher serum copper levels, suggesting a potential relationship between copper metabolism and delayed hematopoietic recovery (Table 1). These findings highlight that copper ion levels may influence stem cell growth and granulocyte recovery post-chemotherapy, implicating copper-induced cell death (cuproptosis) as a contributing factor to hematopoietic impairment in AML patients. This underscores the need for further investigation into the role of copper death pathways in post-chemotherapy recovery dynamics.

**TABLE 1:** Study participant characteristics at enrollment.

Variables	Total (n = 214)	Cohort, median (IQR)		P-value
		Neutropenia_Duration >25 days (n = 107)	Neutropenia_Duration ≤25 days (n = 107)	
Age, Median (Q1,Q3)	36 (26.25, 45)	38 (29, 46)	35 (25, 43.5)	0.36
Gender, n (%)				0.213
Female	124 (58)	57 (53)	67 (63)	
Male	90 (42)	50 (47)	40 (37)	
WBC (×10 ^ 9), Median (Q1,Q3)	17 (11, 25.75)	17 (12, 27)	16 (10, 23)	0.146
Hb (g/L), Median (Q1,Q3)	78.5 (72, 90)	78 (71, 90)	79 (72, 90)	0.857
PLT (×10 ^ 9), Median (Q1,Q3)	143.5 (79, 223.5)	146 (77, 233.5)	143 (83.5, 206.5)	0.714
ELN Score, n (%)				0.696
Adverse	45 (21)	25 (23)	20 (19)	
Favorable	100 (47)	49 (46)	51 (48)	
Intermediate	69 (32)	33 (31)	36 (34)	
Induction therapy, n (%)				0.11
Low intensity	76 (36)	35 (33)	41 (38)	
Others	21 (10)	15 (14)	6 (6)	
Standard therapy	117 (55)	57 (53)	60 (56)	
Status, n (%)				1
CR	183 (86)	92 (86)	91 (85)	
NR	31 (14)	15 (14)	16 (15)	
Serum Copper Level (μmol/L), Median (Q1,Q3)	32 (24.25, 39)	35 (27.5, 44)	28 (21, 35)	<0.001

2017 ELN: 2017 European Leukemia Network; CR: complete remission; CR: complete remission; NR: no response; IQR, interquartile range; WBC, white blood cell; Hb, hemoglobin; PLT, platelet.

### Cuproptosis-related lncRNAs and prognostic model development in AML

**FIGURE 2:**
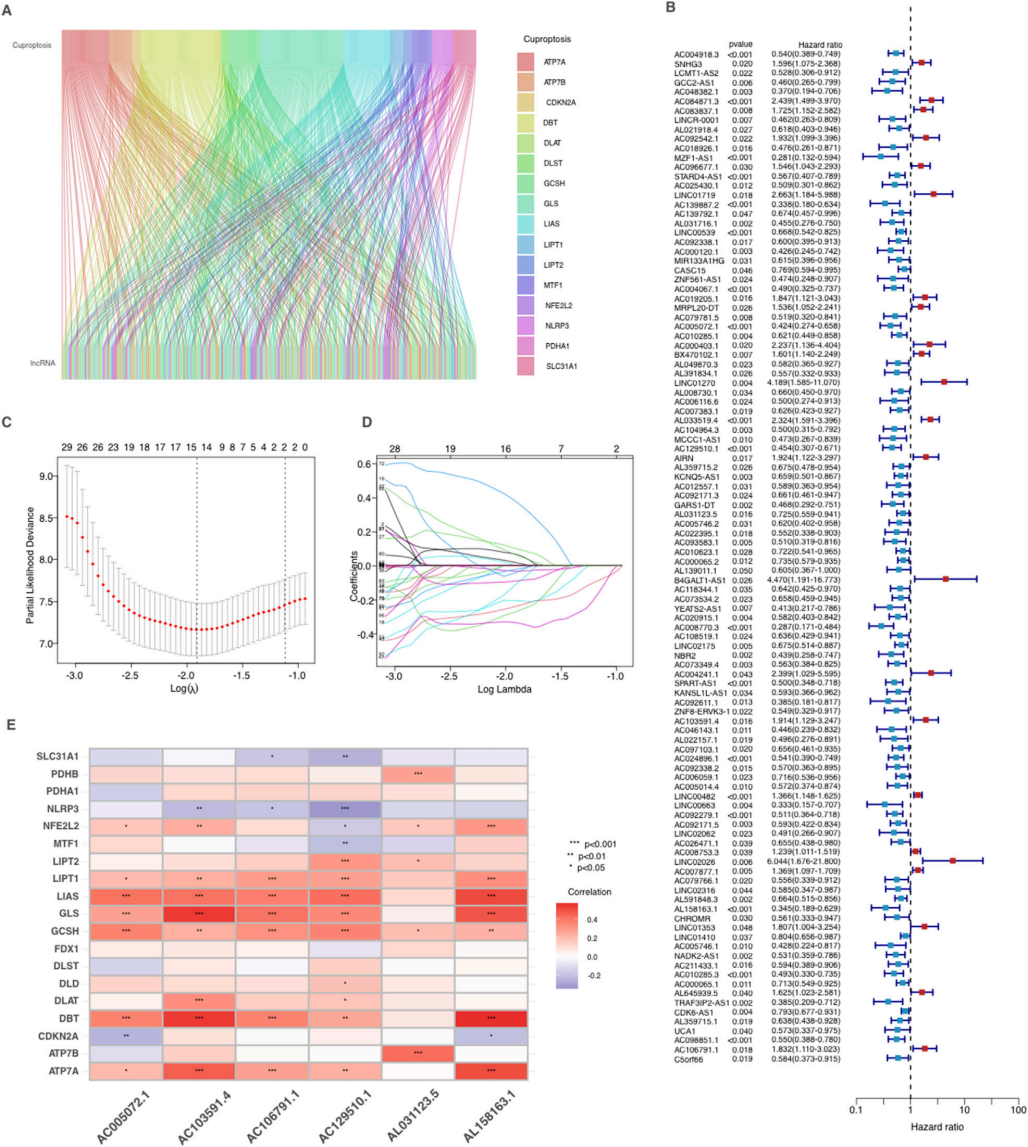
Identification and prognostic analysis of Cuproptosis-related lncRNAs in AML. **(A)** Sankey relationship diagram of cuproptosis genes and their associated lncRNAs. **(B)** Identification of prognostic Cuproptosis-related lncRNAs by univariate Cox regression analysis. **(C–D)** Lasso–Cox regression analysis was performed to develop the prognostic model. **(E)** Heatmap of the correlation between cuproptosis-related genes and 6 prognostic cuproptosis-related lncRNAs. *p < 0.05, **p < 0.01, and ***p < 0.001.

In our study, we investigated lncRNAs associated with cuproptosis in AML from TCGA dataset by filtering cuproptosis-related genes and applying the Pearson correlation analysis. The connections between cuproptosis-related genes and their corresponding lncRNAs were depicted in a Sankey plot (Figure 2A). Subsequently, a analysis of univariate Cox regression was produced to determine the prognostic importance of these lncRNAs. As demonstrated in the forest plot (Figure 2B), 108 lncRNAs were identified as significantly impacting OS of AML patients, with p-values below 0.05. Among these, some lncRNAs had hazard ratios (HR) greater than 1, indicating that they may be associated with poorer prognosis and increased risk of mortality, while others had HR values less than 1, suggesting a potential protective effect and association with improved survival.

For model construction, a cohort of 139 patients was separated into the training group (n = 69) and the testing group (n = 70). The model was built using data from the training group and subsequently validated with the testing group. The clinical characteristics of the AML patients are detailed in Table 2. Crucially, no significant differences in clinical features for the training and validation groups were observed, demonstrating that both groups were well-matched for further analysis.

**TABLE 2:** The clinical basic information of age and gender distribution from TCGA-AML.

Covariates	Type	Total	Test	Train	p value
Age	≤65	99 (71.22%)	46 (66.67%)	53 (75.71%)	0.3218
	>65	40 (28.78%)	23 (33.33%)	17 (24.29%)	
Gender	Female	62 (44.6%)	32 (46.38%)	30 (42.86%)	0.8051
	Male	77 (55.4%)	37 (53.62%)	40 (57.14%)	

To refine the selection of lncRNAs for prognostic modeling, we produced Lasso-Cox regression, which reduced the number of variables by selecting key lncRNAs based on regression coefficient trajectories and cross-validation results (Figures 2C,D). These lncRNAs were further refined using multiple stepwise Cox regression, resulting in a final CRLPM that applied as a robust prognostic tool to predicte patient outcomes in AML. Finally, a heatmap (Figure 2E) was developed to examine the correlations between the selected lncRNAs and the cuproptosis-related genes. Significant correlations were observed between several lncRNAs and key cuproptosis genes, indicating their potential involvement in the regulation of cuproptosis pathways in AML.

### Model validation and risk stratification in AML

**FIGURE 3:**
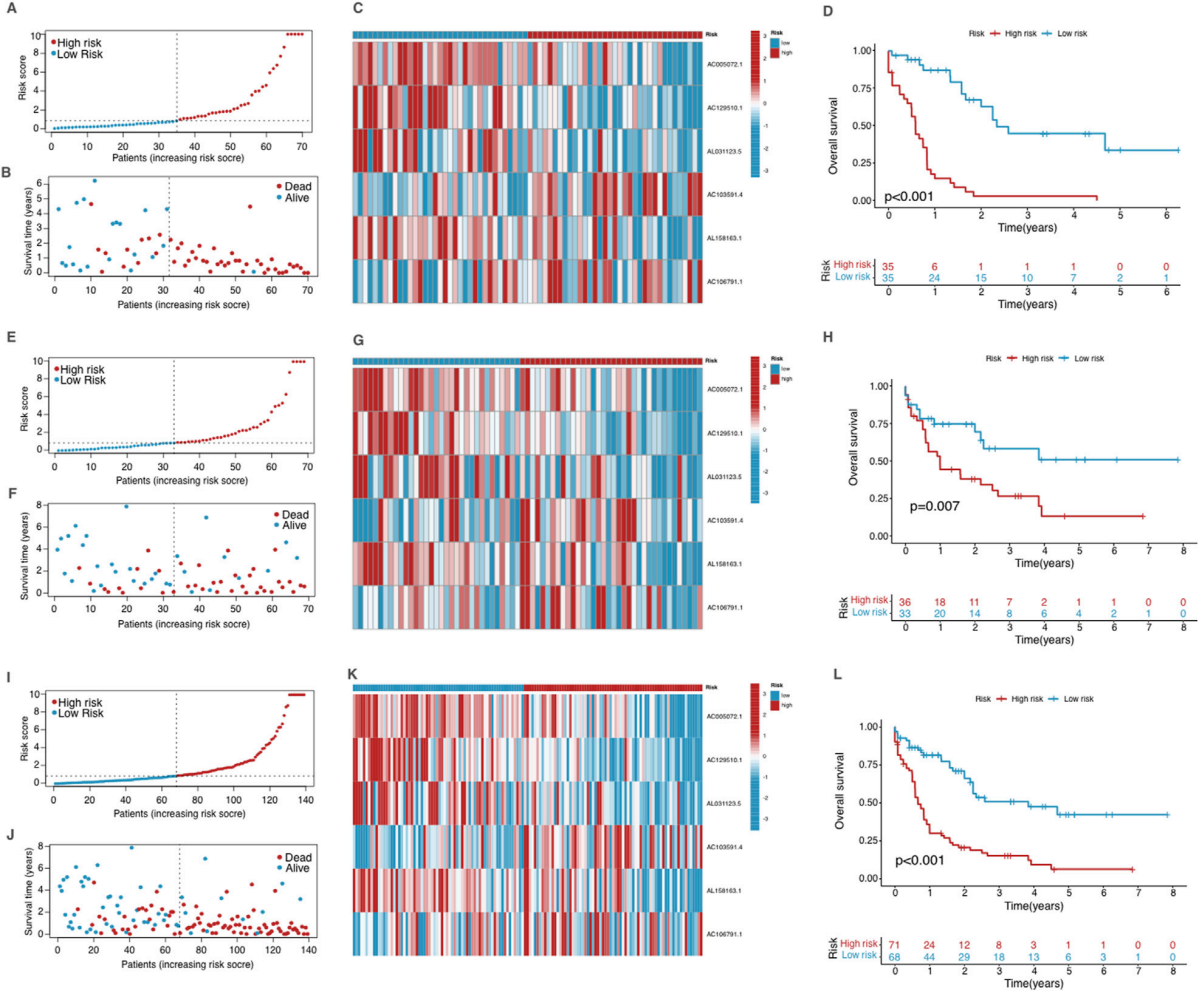
Validation of the prognostic model in the training group **(A–D)**, testing group **(E–H)** and the entire dataset **(I–L)**. **(A, E, I)** The risk score distribution in the corresponding cohort. **(B, F, J)** The survival status in the corresponding cohort. **(C, G, K)** Heatmap of the expression of the prognostic cuproptosis-related lncRNAs in the corresponding cohort. **(D, H, L)** Kaplan–Meier curves for survival analysis for the corresponding cohort.

By setting the median risk score as a cutoff standard, patients were stratified into two groups with high and low risk. The distribution of risk scores is depicted in the risk score plot, clearly differentiating between the two groups (Figure 3A). After stratification, the survival status plot showed that the high-risk group had more deaths and significantly shorter survival times compared to the low-risk group (Figure 3B). The differential expression of cuproptosis-related lncRNAs between two groups was visualized through a heatmap (Figure 3C), with several lncRNAs showing considerable differences between the groups, correlating with their stratified risk levels. The Kaplan-Meier survival analysis was conducted to compare OS between the high-risk and low-risk groups in the training cohort (Figure 3D). The survival curves revealed a significant difference between two risk groups (p < 0.001). Patients with low risk exhibited significantly better OS rates compared to those in the high-risk group.

We validated the model using the testing group and the entire dataset, and the obtained results were consistent with those observed in the training group (Figures 3E–L), showing significant differences in survival outcomes and lncRNA expression profiles across all cohorts. These findings validate the robustness of the CRLPM model in stratifying AML patients by risk, providing consistent prognostic insights across different patient groups based on risk scores and associated lncRNA expression.

### Predictive performance of the CRLPM across expression profiles and clinical subgroups

**FIGURE 4:**
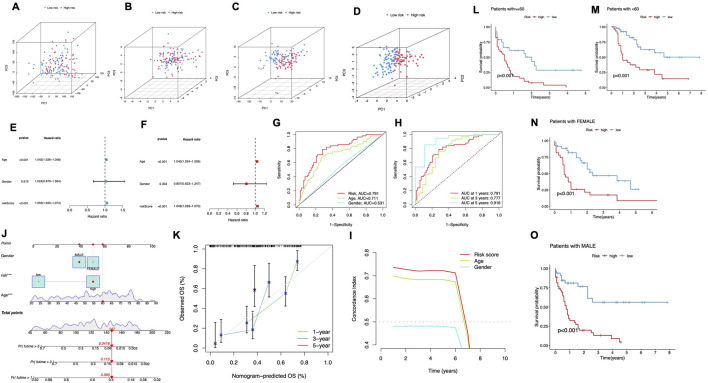
Predictive Performance of the CRLPM. **(A–D)** Principal component analysis (PCA) comparing the high- and low-risk groups based on all genes **(A)**, cuproptosis-related genes **(B)**, cuproptosis-related lncRNAs **(C)** and prognostic lncRNA prognostic markers **(D)**. **(E–F)** The univariate Cox regression analysis **(E)** and the multivariate Cox regression analysis **(F)** of the association between age, gender, risk score and patient outcomes. **(G)** TimeROC curve predicted the 1, 3 and 5-year OS for AML patients. **(H)** ROC demonstrated the predictive accuracy of the risk model was superior to other clinical parameters. **(I)** C-index curve of the risk mode. **(J)** A nomogram combining clinicopathological variables and risk scores predicts 1-, 3-, and 5-years OS in AML patients. **(K)** Calibration curves test the agreement between actual and predicted outcomes at 1, 3, and 5 years. **(L–O)** The Kaplan–Meier curves depicting the OS of patients with different age **(L, M)** and gender **(N, O)**.

PCA was performed to assess the differences across various expression profiles. The PCA based on total gene expression and cuproptosis gene expression showed limited separation between the high- and low-risk groups, with the two groups clustering closely (Figure 4A, B). However, PCA focusing on cuproptosis-related lncRNAs (Figure 4C) revealed a clearer separation between the two groups, indicating that these lncRNAs are more effective in distinguishing between risk categories. The most significant separation was observed when analyzing the six lncRNAs used in the risk model (Figure 4D), which displayed distinct clustering of high- and low-risk groups. In these figures, PC1 accounted for the largest proportion of variance, reflecting the most substantial variation in the data, while PC2 and PC3 contributed to additional but smaller variances. This demonstrates that the selected lncRNAs, particularly the six used in the prognostic model, are highly efficient in stratifying AML patients by risk, with PC1 capturing the primary factor driving this separation. These findings further support the robustness of the risk model in differentiating patient outcomes.

To evaluate the predictive strength of the prognostic model, both univariate and multivariate Cox regression analyses were performed. In the univariate analysis (Figure 4E), significant associations were identified between patient outcomes and both age and risk score, while gender did not exhibit a statistically significant association (p = 0.913), The multivariate Cox regression analysis validated the independent prognostic significance of both age and risk score, confirming their roles as key survival predictors (Figure 4F). ROC curves were plotted for different time points. The model showed strong predictive ability, with AUC values of 0.791 at 1 year, 0.777 at 3 years, and 0.916 at 5 years (Figure 4G). Moreover, the risk score consistently demonstrated superior prognostic accuracy compared to other clinical factors. The multivariate Cox regression analysis validated the independent prognostic significance of both age and risk score, confirming their role as significant predictors of survival (Figure 4F). To further assess the cuproptosis-related lncRNA model’s accuracy in predicting OS, ROC curves were plotted for different survival periods. The model demonstrated robust predictive capability, with a series of AUC values (1 year-0.791, 3 years-0.777, while 5 years-0.916) (Figure 4G). Besides, the risk score consistently showed superior prognostic accuracy when compared to other clinical variables. Specifically, the AUC for the 1-year risk score was 0.791 outperforming other factors like age (AUC = 0.711) and gender (AUC = 0.531) (Figure 4H). Additionally, the C-index for the risk model and age indicated superior prognostic performance over a 6-year period compared to gender, reinforcing its strength as a long-term predictive tool for patient outcomes (Figure 4I). These results align with both the ROC curve Cox and regression analysis findings, demonstrating that the cuproptosis-related lncRNA model is a robust and reliable predictor of survival, reinforcing its strength as a predictive tool for patient outcomes.

A nomogram was developed to quantitatively predict clinical outcomes for AML patients by incorporating the risk score alongside key clinical features such as age and gender (Figure 4J). This tool was designed to estimate individualized survival probabilities at 1–5 years. The nomogram’s performance was validated using calibration plots, which displayed a close match of the predicted survival outcomes and the actual observed data at each time point. This high degree of alignment underscores the accuracy and reliability of the nomogram for predicting patient prognosis over multiple years (Figure 4K).

We stratified patients by age and gender to assess the risk model’s performance. For patients under 60 and those 60 and above, the high-risk group consistently had significantly worse survival outcomes compared to the low-risk group (P < 0.001, Figures 4L,M). Similarly, in both male and female patients, the low-risk group exhibited markedly longer survival times (P < 0.001, Figures 4N,O). These findings confirm the model’s predictive power across different demographic subgroups.

### Biological functions and pathway enrichment analysis of DEGs

To better understand the biological functions and pathways associated with the differentially expressed genes (DEGs) between the high- and low-risk groups, we performed GO and KEGG enrichment analyses. The analysis revealed significant enrichment in various biological processes, cellular components, and molecular functions.

**FIGURE 5:**
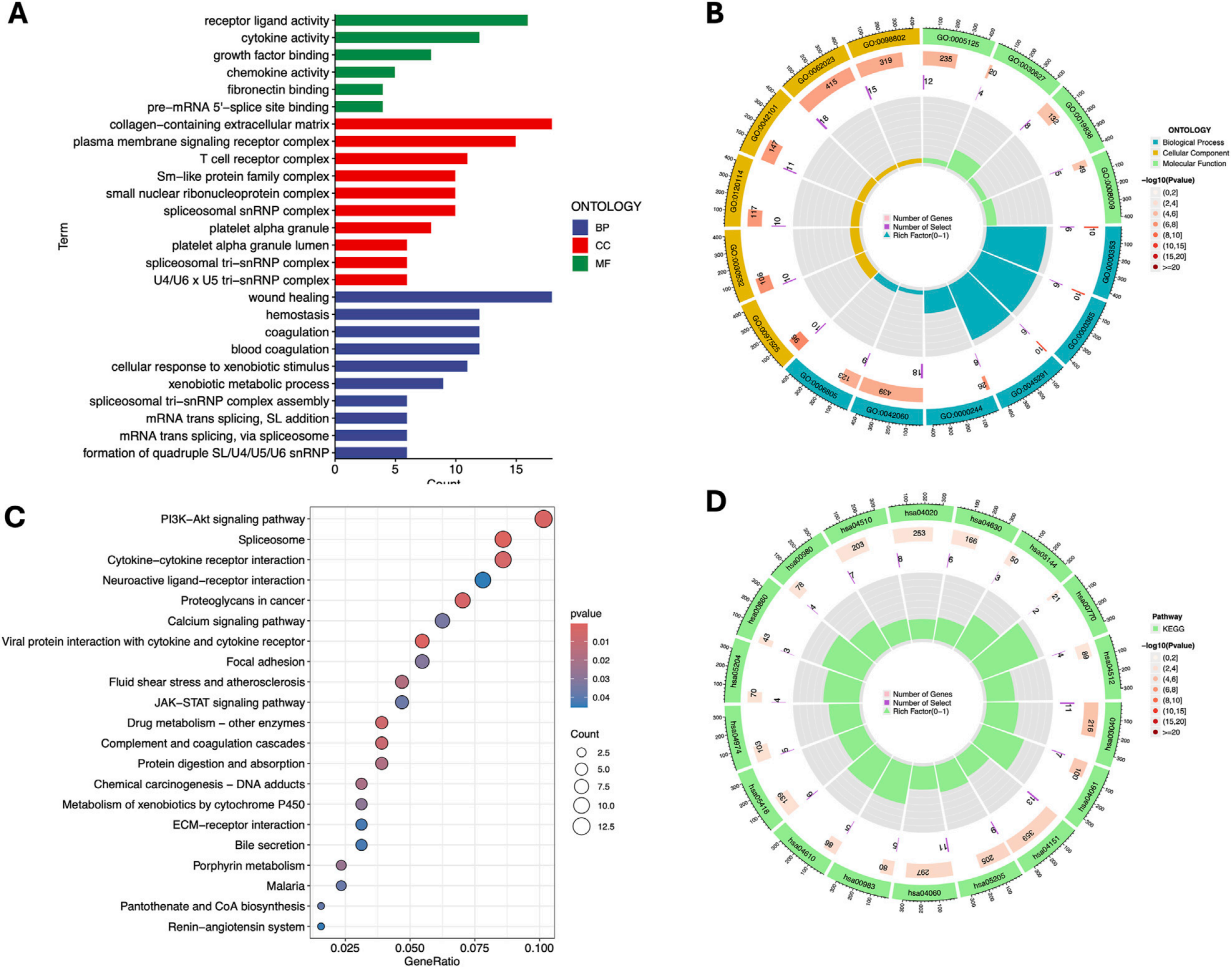
Gene Ontology (GO) and Kyoto Encyclopedia of Genes and Genomes (KEGG) pathway enrichment analysis. Barplot **(A)** and Circle diagram **(B)** display the enriched GO terms. Bubble plot **(C)** and Circle diagram **(D)** presenting the results of enriched KEGG terms. GO term: GO:0062023 - Collagen-containing extracellular matrix; GO:0098802 - Receptor complex; GO:0005125 - Cytokine activity; GO:0030627 - Prefoldin complex; GO:0019838 - Growth factor binding; GO:0008009 - Chemokine activity; GO:0042101 - T cell proliferation; GO:0120114 - Plasma membrane bounded cell projection morphogenesis; GO:0030532 - Small GTPase mediated signal transduction; GO:0097525 - Morphogenesis of a branching epithelium; GO:0006805 - Xenobiotic metabolic process; GO:0042060 - Wound healing; GO:0000244 - Spliceosomal complex assembly; GO:0045291 - Structure morphogenesis; GO:0000365 - mRNA splicing, via spliceosome; GO:0000353 - Regulation of mRNA splicing, via spliceosome. KEGG term: hsa04510 - Focal adhesion; hsa04020 - Calcium signaling pathway; hsa04630 - Jak-STAT signaling pathway; hsa05144 - Malaria; hsa00770 - Pantothenate and CoA biosynthesis; hsa04512 - ECM-receptor interaction; hsa00980 - Metabolism of xenobiotics by cytochrome P450; hsa00860 - Porphyrin and chlorophyll metabolism; hsa05204 - Chemical carcinogenesis; hsa04974 - Protein digestion and absorption; hsa05418 - Fluid shear stress and atherosclerosis; hsa04610 - Complement and coagulation cascades; hsa00983 - Drug metabolism - other enzymes; hsa04060 - Cytokine-cytokine receptor interaction; hsa05205 - Proteoglycans in cancer; hsa04151 - PI3K-Akt signaling pathway; hsa04061 - Viral protein interaction with cytokine and cytokine receptor; hsa03040 - Spliceosome.

In the BP category, the DEGs were significantly enriched in processes such as blood coagulation, wound healing, and cellular response to xenobiotic stimuli, indicating their involvement in immune response and tissue repair. For the CC term, the DEGs were associated with cellular structures including the spliceosomal complex, platelet alpha granule lumen, and collagen-containing extracellular matrix, suggesting a role in signal transduction and extracellular matrix organization, In addition, the T cell receptor complex and membrane signaling receptor complex in the CC category, as well as cytokine activity, receptor ligand activity, and chemokine activity in the MF category, emphasize their importance in immune regulation and cellular communication (Figures 5A,B).

KEGG pathway analysis further revealed significant associations with critical pathways, such as the PI3K-Akt signaling pathway, spliceosome, and cytokine-cytokine receptor interaction, which are likely to play crucial roles in the disease mechanism. Additionally, pathways like JAK-STAT signaling, focal adhesion and drug metabolism were identified, implicating their involvement in cell survival, proliferation, drug resistance and inflammation (Figures 5C,D).

### Immune cell infiltration and immunotherapy response prediction

**FIGURE 6:**
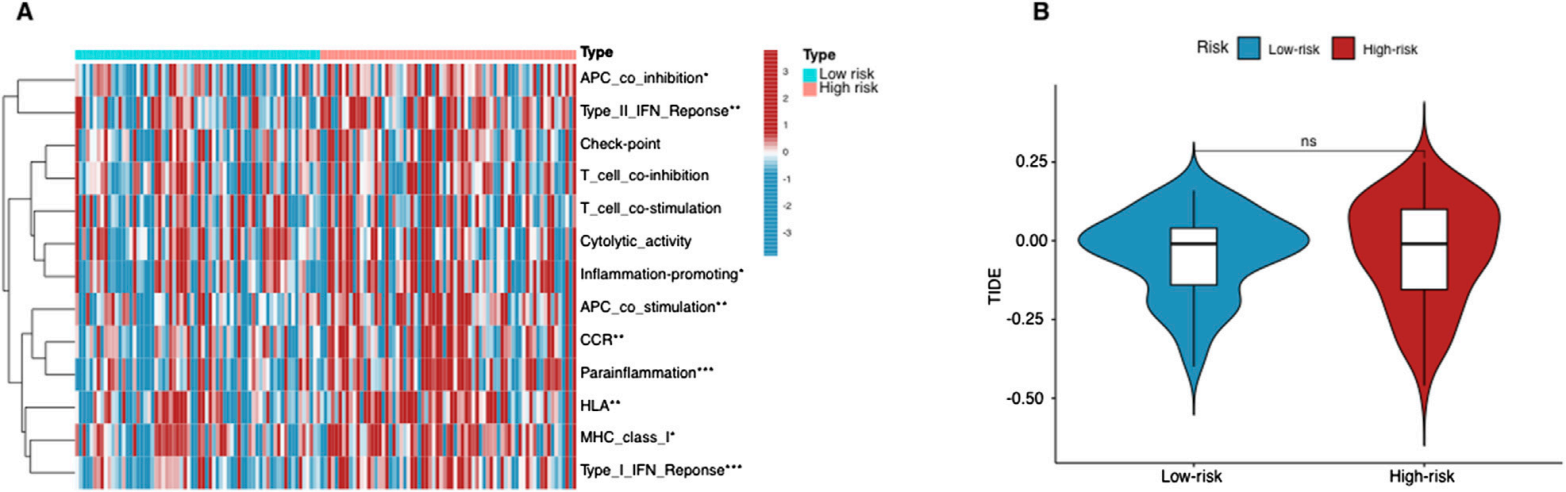
Immunological landscape in AML patients. **(A)** Heatmap of the tumor-infiltrating lymphocytes based on ssGSEA algorithms among the high- and low-risk groups in AML. **(B)** The comparison of TIDE prediction scores between the high- and low-risk groups. *p < 0.05, **p < 0.01, and ***p < 0.001.

Using the ssGSEA algorithm, we compared immune-related pathways between the high-risk and low-risk groups. The heatmap (Figure 6A) revealed significant differences in several key immune regulatory pathways, including APC co-inhibition/stimulation, IFN Response, MHC Class I, parainflammation, and inflammation-promoting pathways. These findings suggest that the immune microenvironment differs notably between the two groups, with high-risk patients showing altered immune regulation, potentially facilitating tumor immune evasion. On the other hand, T cell-related functions, such as T cell co-stimulation or inhibition, and cytolytic activity, showed no significant differences between the two groups. This indicates that the overall T cell functionality, particularly in terms of immune activation and cytolytic responses, remains similar across both risk groups.

The lack of significant differences in T cell-related immune activities aligns with the TIDE analysis results (Figure 6B). The TIDE scores, which assess tumor immune evasion potential, did not show a statistically significant difference between the two groups. This suggests that T cell-mediated immune escape mechanisms are not the primary drivers of the differences observed in immune response between these groups. Instead, the divergence in immune regulation appears to be more associated with APC function, cytokine responses, and inflammatory processes, rather than T cell-specific mechanisms.

To further elucidate the composition of the tumor immune microenvironment, we additionally employed the CIBERSORT algorithm, which deconvolutes bulk transcriptomic data to estimate the relative proportions of different immune cell types. The CIBERSORT results showed variations in specific immune cell populations—particularly those involved in antigen presentation (e.g., dendritic cells) and inflammatory responses (e.g., certain macrophage subtypes)—between high-risk and low-risk patients. However, consistent with the ssGSEA findings, T cell subsets (including CD8^+^ T cells) did not significantly differ in abundance between the two groups. These observations reinforce the notion that while certain pathways and cell types critical for immune regulation and inflammation diverge between risk groups, T cell infiltration and cytolytic function remain relatively unchanged.

### Tumor mutational burden and its association with risk of AML

**FIGURE 7:**
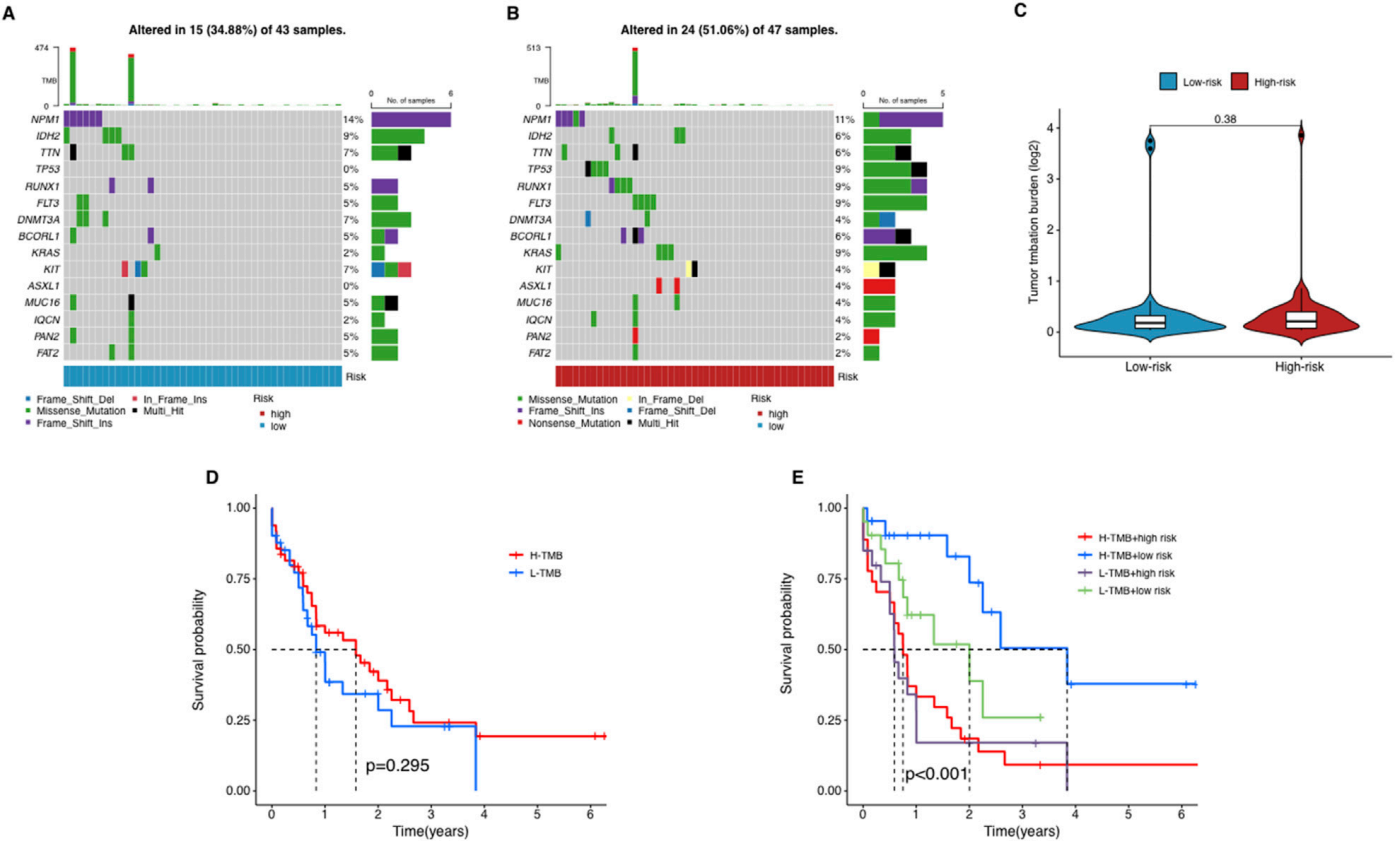
The relationship between tumor mutation burden (TMB) and risk score. **(A–C)** The waterfall plot of mutant genes in the high- **(A)** and low-risk group **(B)** in AML patients. **(D)** The Kaplan–Meier curves depicting the OS of patients with different TMB groups. **(E)** The Kaplan–Meier curves depicting the OS of patients with different TMB and risk groups.

We used waterfall plots to visualize the mutation landscape between two risk groups. The mutation frequencies of TP53 and KRAS were particularly prominent in the high-risk group (Figures 7A,B). TP53 is a widely used tumor suppressor gene, while KRAS is an oncogene associated with tumor aggressiveness. In contrast, most other genes did not exhibit significant differences between two groups. It suggests that mutations in TP53 and KRAS probably are key factors contributing to the poor prognosis of high-risk AML patients.

To explore the potential role of TMB in AML, we collected somatic mutation data from AML samples and calculated TMB scores. Our analysis revealed no significant differences in TMB scores between the groups (Figure 7C), suggesting that TMB does not correlate significantly with risk stratification in AML. Further, patients were categorized into high-TMB and low-TMB groups based on the median TMB score. Survival analysis indicated that TMB alone does not significantly influence OS among AML patients, as no notable differences were observed between the high-TMB and low-TMB groups (Figure 7D). In a subsequent combined survival analysis that included both TMB and risk scores, it was found that patients in the high-risk, low-TMB category had the poorest survival outcomes, while those in the low-risk, low-TMB category exhibited the best survival rates (Figure 7E). These findings imply that integrating lncRNA profiles with TMB and risk scores might offer a more detailed prognostic assessment for AML patients.

### Drug sensitivity analysis and individualized treatment potential of CRLPM in AML

**FIGURE 8:**
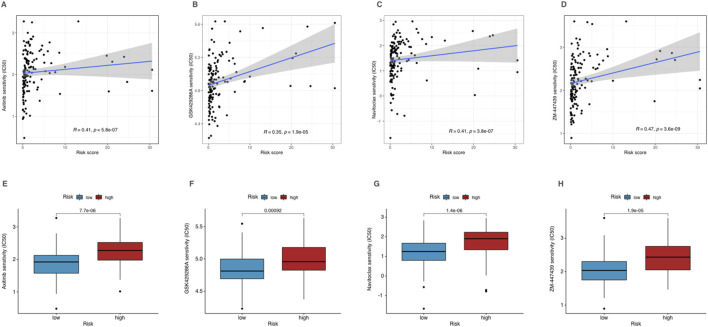
Drug sensitivity analysis of high- and low-risk patients in AML. **(A–D)** Correlation analysis of corresponding drug IC50 and risk scores. **(E–H)** The IC50 of corresponding drugs in high- and low-risk patients in AML.

We explored the correlation between risk scores and IC50 values of various chemotherapeutic drugs to assess the individualized treatment potential of the CRLPM in AML. The results showed that among all drugs, Axitinib, GSK429286A, Navitoclax, and ZM-447439 exhibited significant difference in sensitivity for group with different level of risk (p < 0.01) (Figures 8A–D). These drugs displayed higher IC50 values for high-risk group, suggesting lower sensitivity to these agents (Figures 8E–H). This suggests that AML patients with higher CRLPM scores, which are associated with copper-induced cell death mechanisms, may not respond effectively to these drugs. The high drug concentrations required to achieve therapeutic effects in these patients may lead to increased toxicity and poor treatment outcomes.

## Discussion

### Model effectiveness and clinical applications

The results of lncRNAs associated with cuproptosis suggested that lncRNAs have a significant impact on the prognosis of AML patients. By identifying lncRNAs that were strongly associated with OS, we developed a prediction model by statistical methods such as Lasso-Cox regression to stratify patients into risk groups with different survival outcomes. CRLPM demonstrated robust predictive accuracy for AML survival outcomes, supported by AUC values across different time points. This predictive strength aligns with previous studies where lncRNA-based models successfully stratified AML patients into distinct risk categories, reinforcing the prognostic role of lncRNAs in AML. It may enable high-risk patients to receive more aggressive treatment modalities or therapies targeting lncRNA activity or induction of cuproptosis while avoiding overtreatment of low-risk patients. This stratification also highlighted the potential of these lncRNAs as therapeutic targets. The robust performance aligns with findings from previous research where lncRNA-based models stratified AML patients effectively, reinforcing lncRNAs’ role as prognostic indicators in hematological malignancies like AML.

Clinically, CRLPM could serve as a personalized tool to aid in decision-making for high-risk patients, potentially complementing traditional prognostic markers like FLT3 (Jalte et al., 2023) or TP53 mutations (Shin, 2023), which are commonly used in clinical settings (Springer et al., 2024). AML treatment usually applies to a strong chemotherapy regimen to reduce leukemia cells in the bone marrow through induction therapy. By distinguishing high-risk patients who may have lower sensitivity to certain chemotherapies, CRLPM can support treatment personalization, potentially reducing adverse effects for those likely to respond poorly.

Furthermore, the identification of copper metabolism pathways within CRLPM indicates its utility as an alternative biomarker for therapies targeting cuproptosis mechanisms, opening avenues for copper-targeted treatment strategies in high-risk AML patients. The classic induction therapy regimen includes the “7 + 3″ regimen, which is 7 consecutive days of intravenous injection of cytarabine combined with 3 days of anthracyclines (such as daunorubicin or aclarubicin) (Kantarjian et al., 2021). By analyzing the sensitivity of chemotherapy drugs in the database, we found that only four drugs (Axitinib, GSK429286A, Navitoclax and ZM-447439) showed higher sensitivity in low-risk patients through mechanisms such as inhibiting angiogenesis, targeting Rho kinase, inhibiting Bcl-2 and inhibiting Aurora kinase (Gross-Goupil et al., 2013; Fayed et al., 2023; Mohamad Anuar et al., 2020; Gadea and Ruderman, 2005), Although their clinical use in the treatment of AML is still mostly experimental, these drugs have the therapeutic potential to be advanced to preclinical or clinical studies in the future. Additionally, the use of Axitinib, GSK429286A, Navitoclax, and ZM-447439 should be avoided in these patients, as the high IC50 values indicate poor efficacy and the potential for severe adverse effects due to the need for higher dosages. Therefore, for high-risk AML patients, alternative therapies like hematopoietic stem cell transplantation (HSCT) or CAR-T therapy could be more appropriate (Wang Q. Y. et al., 2024; Marofi et al., 2021). These findings suggest that using CRLPM for risk stratification can guide treatment decisions in AML, helping to identify patients who may benefit more from alternative therapies while reducing the risk of adverse outcomes associated with ineffective chemotherapy.

### Biological role of cuproptosis-related lncRNAs in AML

lncRNAs have been implicated in various malignancies, affecting cell proliferation, apoptosis, and metastasis (Zangouei et al., 2023; Heydarnezhad Asl et al., 2022). Our results extend these findings to cuproptosis, suggesting that lncRNAs may play a key role in mediating the response of AML cells to copper-induced stress. Similarly, current findings suggest that XIST and MEG3 could potentially modulate cuproptosis in AML cells by influencing copper metabolism pathways (Xie et al., 2023; Springer et al., 2024). For instance, XIST has been shown to influence survival by modulating downstream molecules like miRNAs that regulate copper metabolism-related enzymes in AML. XIST downregulates miR-142-5p, which in turn upregulates enzymes like phosphofructokinase (PFKP), enhancing glycolysis and promoting cell proliferation (Jiang et al., 2024). This upregulation of metabolic enzymes creates a cellular environment that can tolerate higher oxidative stress, allowing AML cells to evade copper-induced cell death. Similarly, MEG3 has been shown to promote cell death in AML through its interaction with TP53 and other apoptotic pathways, further supporting its tumor-suppressive role. Downregulation of MEG3 correlates with poor prognosis in AML, likely due to decreased sensitivity to oxidative stress and reduced activation of cuproptosis. MEG3 might act by modulating copper-related enzymatic pathways, potentially leading to copper accumulation in cancer cells and triggering cuproptosis (Zhang et al., 2022). In this study, our model identified six understudied but critical lncRNAs and explored their important prognostic value in AML. Our results innovatively revealed that these lncRNAs may regulate important signaling pathways in response to cuproptosis. Future therapeutic functional studies are needed to elucidate the exact mechanism of action of these lncRNAs and their potential as therapeutic targets for AML.

### Influence on immune infiltration and tumor microenvironment

CRLPM results also indicated notable interactions between copper metabolism and the AML tumor microenvironment, particularly in immune infiltration. Disorder in copper homeostasis have been observed in several cancers (Lelievre et al., 2020; Bian et al., 2023),Although it has attracted widespread attention in research, there are currently no drugs specifically targeting copper apoptosis in clinical or preclinical applications. Compared with solid tumors, AML is extremely challenging due to its high heterogeneity and the involvement of both hematopoietic stem and progenitor cells in its pathogenesis (Chen et al., 2022). Different from solid tumor microenvironments, AML develops in the bone marrow microenvironment, where cancer stem cells (CSCs) and leukemia stem cells (LSCs) play a key role in the initiation, development, metastasis, and relapse of the disease. These LSCs have self-renewal capabilities and are resistant to traditional chemotherapy, making them a key factor in AML relapse and poor prognosis (Pimenta et al., 2021; Thomas and Majeti, 2017). The differential expression and enrichment analysis in this study revealed several enriched signaling pathways that are highly relevant to cancer stem cell biology: PI3K-Akt signaling, which regulates cell proliferation and growth, is also important for the self-renewal and homeostasis maintenance of leukemic stem cells in AML (Gu et al., 2024). In addition, JAK-STAT signaling pathway, ECM-receptor interaction and focal adhesion pathways were also proven to be significantly relevant to cancer stem cells and the bone marrow microenvironment (Huang et al., 2021; Zanetti and Krause, 2020). The results also showed that cytokine and receptor activity were enriched in AML, further validating the role of inflammatory signals such as IL-6 or TNF-α in the development of cancer stem cells. These findings also emphasize the complex link between copper metabolism and immune response in AML, suggesting that immune modulation could be a viable therapeutic strategy for high-risk AML patients. CRLPM may also serve as a biomarker for identifying candidates for immunotherapy, particularly treatments targeting immune checkpoints and antigen presentation pathway.

Our research explored differences in the immune microenvironment between low-risk and high-risk groups, but no notable differences were observed for T cell-related functions, indicating that T cell activity remains stable across risk groups. This suggests that immune evasion in the high-risk group may not be primarily driven by T cell dysfunction, but rather by disruptions in antigen presentation and other non-T cell immune mechanisms. This hypothesis is consistent with the TIDE analysis, which showed no significant differences in TIDE scores between the two groups. In the high-risk group, tumors may evade immune responses by inhibiting antigen presentation and reducing immune cell infiltration. Given these findings, targeting antigen presentation pathways (e.g., MHC Class I) and reducing chronic inflammation may represent more effective strategies for treating high-risk patients, rather than therapies solely focused on enhancing T cell activity.

TP53 and KRAS were confirmed to have significantly high mutation frequencies in the high-risk group, suggesting that therapeutics targeting these two genes may improve patient prognosis. Although our analysis showed that the TMB score did not significantly affect patient OS, when TMB was grouped together with our model, it had a better distinction for patient prognosis, indicating that therapies targeting these two genes have the potential to be adjuvant treatments, but making such a decision still requires careful and comprehensive consideration.

### Study limitations and future directions

While CRLPM presents significant potential, several limitations warrant consideration. First, the model’s reliance on publicly available datasets introduces potential bias stemming from demographic or population-specific factors. This highlights the need for multi-center, prospective studies that include diverse patient populations to minimize sampling bias and better capture the heterogeneity of AML. Second, the lack of detailed AML subtype stratification restricts CRLPM’s current applicability, given AML’s diverse genetic and clinical profiles (e.g., FLT3, NPM1, IDH mutations, etc.). Future investigations should therefore include larger cohorts and incorporate molecular subtypes to validate CRLPM in a variety of AML subsets.

Moreover, although our analyses focused on cuproptosis, the intricate interplay between copper-induced cell death and other metabolic or cell-death pathways (e.g., ferroptosis, pyroptosis, apoptosis) remains incompletely characterized. Further research dissecting these interactions—including synergy or antagonism among different forms of regulated cell death—could refine CRLPM’s predictive accuracy and expand its mechanistic scope. Additionally, functional experiments are necessary to validate the direct link between identified lncRNAs and cuproptosis, further solidifying the mechanistic foundation of the model. Such studies may involve *in vitro* assays (e.g., siRNA knockdown or CRISPR-Cas9 editing of candidate lncRNAs) to observe changes in copper-dependent cytotoxicity, as well as *in vivo* xenograft models to evaluate disease progression and therapeutic response.

Looking forward, *in vitro* and *in vivo* experiments investigating the interaction of lncRNAs with copper-induced cell death in AML cells will be essential. These efforts may elucidate the precise molecular circuits through which lncRNAs modulate cuproptosis, potentially revealing new therapeutic targets or combination strategies. Integrating single-cell sequencing data and high-resolution imaging could also uncover cell-to-cell heterogeneity in CRLPM activity, leading to more personalized risk assessments and treatment strategies. Finally, combining CRLPM with established prognostic biomarkers or immunotherapy response predictors (e.g., TIDE, CIBERSORT) may yield a multifaceted approach that accounts for both metabolic vulnerability and immune microenvironment dynamics. Altogether, these directions will help establish CRLPM not only as a robust prognostic marker but also as a foundation for new precision medicine strategies in AML.

## Conclusion

In summary, we established a cuproptosis-associated long noncoding RNA prognostic model (CRLPM) for AML treatment. CRLPM effectively stratified patients into high-risk and low-risk groups and accurately predicted OS, providing guidance and reference for clinical treatment and administration strategy. T-cell activity-based therapies and gene therapies targeting TP53 mutations may not be effective in treatment. High-risk patients may have reduced sensitivity to specific chemotherapies, including axitinib, GSK429286A, Navitoclax, and ZM-447439. It indicated that patients defined as high-risk groups by CRLPM should avoid using these drugs in treatment to improve therapeutic activity and reduce side effects. Therefore, the CRLPM provides valuable guidance for personalized potential treatment strategies and prognosis for AML based on risk level. CRLPM could improve the clinical outcomes of AML by combining risk stratification and formulating treatments accordingly.

## Data Availability

The raw data supporting the conclusions of this article will be made available by the authors, without undue reservation.

## References

[B1] Abaza Y. McMahon C. Garcia J. S. (2024). Advancements and challenges in the treatment of AML. Am. Soc. Clin. Oncol. Educ. Book 44 (3), e438662. 10.1200/edbk_438662 38662975

[B2] Albrecht T. A. (2014). Physiologic and psychological symptoms experienced by adults with acute leukemia: an integrative literature review. Oncol. Nurs. Forum 41 (3), 286–295. 10.1188/14.ONF.286-295 24769593 PMC4368444

[B3] Ali A. M. Salih G. F. (2023). Molecular and clinical significance of FLT3, NPM1, DNMT3A and TP53 mutations in acute myeloid leukemia patients. Mol. Biol. Rep. 50 (10), 8035–8048. 10.1007/s11033-023-08680-2 37540457

[B4] Bian C. Zheng Z. Su J. Chang S. Yu H. Bao J. (2023). Copper homeostasis and cuproptosis in tumor pathogenesis and therapeutic strategies. Front. Pharmacol. 14, 1271613. 10.3389/fphar.2023.1271613 37767404 PMC10520736

[B5] Chen Y. Li J. Xu L. Gaman M. A. Zou Z. (2022). The genesis and evolution of acute myeloid leukemia stem cells in the microenvironment: from biology to therapeutic targeting. Cell Death Discov. 8 (1), 397. 10.1038/s41420-022-01193-0 36163119 PMC9513079

[B6] Chen Y.-F. Li J. Xu L.-L. Găman M.-A. Zou Z.-Y. (2023). Allogeneic stem cell transplantation in the treatment of acute myeloid leukemia: an overview of obstacles and opportunities. World J. Clin. Cases 11 (2), 268–291. 10.12998/wjcc.v11.i2.268 36686358 PMC9850970

[B7] Fayed H. S. Bakleh M. Z. Ashraf J. V. Howarth A. Ebner D. Al Haj Zen A. (2023). Selective ROCK inhibitor enhances blood flow recovery after hindlimb ischemia. Int. J. Mol. Sci. 24 (19), 14410. 10.3390/ijms241914410 37833857 PMC10572734

[B8] Gadea B. B. Ruderman J. V. (2005). Aurora kinase inhibitor ZM447439 blocks chromosome-induced spindle assembly, the completion of chromosome condensation, and the establishment of the spindle integrity checkpoint in Xenopus egg extracts. Mol. Biol. Cell 16 (3), 1305–1318. 10.1091/mbc.e04-10-0891 15616188 PMC551494

[B9] Gao J. Wang F. Wu P. Chen Y. Jia Y. (2020). Aberrant LncRNA expression in leukemia. J. Cancer 11 (14), 4284–4296. 10.7150/jca.42093 32368311 PMC7196264

[B10] Gourvest M. Brousset P. Bousquet M. (2019). Long noncoding RNAs in acute myeloid leukemia: functional characterization and clinical relevance. Cancers (Basel) 11 (11), 1638. 10.3390/cancers11111638 31653018 PMC6896193

[B11] Gross-Goupil M. Francois L. Quivy A. Ravaud A. (2013). Axitinib: a review of its safety and efficacy in the treatment of adults with advanced renal cell carcinoma. Clin. Med. Insights Oncol. 7, 269–277. 10.4137/CMO.S10594 24250243 PMC3825605

[B12] Gu H. Chen C. Hou Z. S. He X. D. Xie S. Ni J. (2024). PI3Kγ maintains the self-renewal of acute myeloid leukemia stem cells by regulating the pentose phosphate pathway. Blood 143 (19), 1965–1979. 10.1182/blood.2023022202 38271660 PMC11103183

[B13] Heydarnezhad Asl M. Pasban Khelejani F. Bahojb Mahdavi S. Z. Emrahi L. Jebelli A. Mokhtarzadeh A. (2022). The various regulatory functions of long noncoding RNAs in apoptosis, cell cycle, and cellular senescence. J. Cell Biochem. 123 (6), 995–1024. 10.1002/jcb.30221 35106829

[B14] Huang J. Zhang L. Wan D. Zhou L. Zheng S. Lin S. (2021). Extracellular matrix and its therapeutic potential for cancer treatment. Signal Transduct. Target Ther. 6 (1), 153. 10.1038/s41392-021-00544-0 33888679 PMC8062524

[B15] Iasonos A. Schrag D. Raj G. V. Panageas K. S. (2008). How to build and interpret a nomogram for cancer prognosis. J. Clin. Oncol. 26 (8), 1364–1370. 10.1200/JCO.2007.12.9791 18323559

[B16] Izadirad M. Jafari L. James A. R. Unfried J. P. Wu Z. X. Chen Z. S. (2021). Long noncoding RNAs have pivotal roles in chemoresistance of acute myeloid leukemia. Drug Discov. Today 26 (7), 1735–1743. 10.1016/j.drudis.2021.03.017 33781951

[B17] Jalte M. Abbassi M. El Mouhi H. Daha Belghiti H. Ahakoud M. Bekkari H. (2023). FLT3 mutations in acute myeloid leukemia: unraveling the molecular mechanisms and Implications for targeted therapies. Cureus 15 (9), e45765. 10.7759/cureus.45765 37872917 PMC10590537

[B18] Jiang P. Gu S. Pan D. Fu J. Sahu A. Hu X. (2018). Signatures of T cell dysfunction and exclusion predict cancer immunotherapy response. Nat. Med. 24 (10), 1550–1558. 10.1038/s41591-018-0136-1 30127393 PMC6487502

[B19] Jiang Z. Liu T. Wang Y. Li J. Guo L. (2024). Effect of lncRNA XIST on acute myeloid leukemia cells via miR-142-5p-PFKP axis. Hematology 29 (1), 2306444. 10.1080/16078454.2024.2306444 38305210

[B20] Kantarjian H. M. Kadia T. M. DiNardo C. D. Welch M. A. Ravandi F. (2021). Acute myeloid leukemia: treatment and research outlook for 2021 and the MD Anderson approach. Cancer 127 (8), 1186–1207. 10.1002/cncr.33477 33734442 PMC12084862

[B21] Lelievre P. Sancey L. Coll J. L. Deniaud A. Busser B. (2020). The multifaceted roles of copper in cancer: a trace metal element with dysregulated metabolism, but also a target or a bullet for therapy. Cancers (Basel) 12 (12), 3594. 10.3390/cancers12123594 33271772 PMC7760327

[B22] Li Y. J. Li H. Y. Zhang Q. Wei S. L. (2022). The prognostic value and immune landscape of a cuproptosis-related lncRNA signature in head and neck squamous cell carcinoma. Front. Genet. 13, 942785. 10.3389/fgene.2022.942785 35942287 PMC9356288

[B23] Marofi F. Rahman H. S. Al-Obaidi Z. M. J. Jalil A. T. Abdelbasset W. K. Suksatan W. (2021). Novel CAR T therapy is a ray of hope in the treatment of seriously ill AML patients. Stem Cell Res. and Ther. 12 (1), 465. 10.1186/s13287-021-02420-8 34412685 PMC8377882

[B24] Mattick J. S. Amaral P. P. Carninci P. Carpenter S. Chang H. Y. Chen L. L. (2023). Long non-coding RNAs: definitions, functions, challenges and recommendations. Nat. Rev. Mol. Cell Biol. 24 (6), 430–447. 10.1038/s41580-022-00566-8 36596869 PMC10213152

[B25] Mayakonda A. Lin D. C. Assenov Y. Plass C. Koeffler H. P. (2018). Maftools: efficient and comprehensive analysis of somatic variants in cancer. Genome Res. 28 (11), 1747–1756. 10.1101/gr.239244.118 30341162 PMC6211645

[B26] Mer A. S. Lindberg J. Nilsson C. Klevebring D. Wang M. Grönberg H. (2018). Expression levels of long non-coding RNAs are prognostic for AML outcome. J. Hematol. and Oncol. 11 (1), 52. 10.1186/s13045-018-0596-2 29625580 PMC5889529

[B27] Mishra S. Liu J. Chai L. Tenen D. G. (2022). Diverse functions of long noncoding RNAs in acute myeloid leukemia: emerging roles in pathophysiology, prognosis, and treatment resistance. Curr. Opin. Hematol. 29 (1), 34–43. 10.1097/MOH.0000000000000692 34854833 PMC8647777

[B28] Mohamad Anuar N. N. Nor Hisam N. S. Liew S. L. Ugusman A. (2020). Clinical review: Navitoclax as a pro-apoptotic and anti-fibrotic agent. Front. Pharmacol. 11, 564108. 10.3389/fphar.2020.564108 33381025 PMC7768911

[B29] Pan J. Q. Zhang Y. Q. Wang J. H. Xu P. Wang W. (2017). lncRNA co-expression network model for the prognostic analysis of acute myeloid leukemia. Int. J. Mol. Med. 39 (3), 663–671. 10.3892/ijmm.2017.2888 28204819 PMC5360362

[B30] Pimenta D. B. Varela V. A. Datoguia T. S. Caraciolo V. B. Lopes G. H. Pereira W. O. (2021). The bone marrow microenvironment mechanisms in acute myeloid leukemia. Front. Cell Dev. Biol. 9, 764698. 10.3389/fcell.2021.764698 34869355 PMC8639599

[B31] Shi X. Huang F. Le X. Li X. Huang K. Liu B. (2017). A practical prognostic lncRNA signature for lung squamous cell carcinoma. Transl. Med. Commun. 2 (1), 7. 10.1186/s41231-017-0016-6

[B32] Shin D. Y. (2023). TP53 mutation in acute myeloid leukemia: an old foe revisited. Cancers (Basel) 15 (19), 4816. 10.3390/cancers15194816 37835510 PMC10571655

[B33] Siegel R. L. Giaquinto A. N. Jemal A. (2024). Cancer statistics, 2024. CA A Cancer J. Clin. 74 (1), 12–49. 10.3322/caac.21820 38230766

[B34] Springer C. Humayun D. Skouta R. (2024). Cuproptosis: unraveling the mechanisms of copper-induced cell death and its implication in cancer therapy. Cancers (Basel) 16 (3), 647. 10.3390/cancers16030647 38339398 PMC10854864

[B35] Statello L. Guo C.-J. Chen L.-L. Huarte M. (2020). Gene regulation by long non-coding RNAs and its biological functions. Nat. Rev. Mol. Cell Biol. 22 (2), 96–118. 10.1038/s41580-020-00315-9 33353982 PMC7754182

[B36] Thomas D. Majeti R. (2017). Biology and relevance of human acute myeloid leukemia stem cells. Blood 129 (12), 1577–1585. 10.1182/blood-2016-10-696054 28159741 PMC5364335

[B37] Tian M. Gong W. Guo J. (2019). Long non-coding RNA SNHG1 indicates poor prognosis and facilitates disease progression in acute myeloid leukemia. Biol. Open 8 (10), bio046417. 10.1242/bio.046417 31615767 PMC6826290

[B38] Tsvetkov P. Coy S. Petrova B. Dreishpoon M. Verma A. Abdusamad M. (2022). Copper induces cell death by targeting lipoylated TCA cycle proteins. Science 375 (6586), 1254–1261. 10.1126/science.abf0529 35298263 PMC9273333

[B39] WangQ. Y.HanY. F.LiY. H.WangQ. Y.ZhuJ. Y.DongY. J. (2024b). A novel prognostic scoring system for AML patients undergoing allogeneic hematopoietic stem cell transplantation with real world validation. J. Adv. Res. 10.1016/j.jare.2024.09.014 39299605

[B40] Wang X. Sun H. Dong Y. Huang J. Bai L. Tang Z. (2024a). Development and validation of a cuproptosis-related prognostic model for acute myeloid leukemia patients using machine learning with stacking. Sci. Rep. 14 (1), 2802. 10.1038/s41598-024-53306-7 38307903 PMC10837443

[B41] Watts J. Nimer S. (2018). Recent advances in the understanding and treatment of acute myeloid leukemia. F1000Res 7, F1000 Faculty Rev-1196. 10.12688/f1000research.14116.1 PMC6081972 30135719

[B42] Wu M. Li A. Zhang T. Ding W. Wei Y. Wan C. (2024). The novel prognostic analysis of AML based on ferroptosis and cuproptosis related genes. J. Trace Elem. Med. Biol. 86, 127517. 10.1016/j.jtemb.2024.127517 39270538

[B43] Xie J. Yang Y. Gao Y. He J. (2023). Cuproptosis: mechanisms and links with cancers. Mol. Cancer 22 (1), 46. 10.1186/s12943-023-01732-y 36882769 PMC9990368

[B44] Yao H. Liu P. Yao L. Li X. (2024). Establishment of disulfidptosis-related LncRNA signature as biomarkers in colon adenocarcinoma. Cancer Cell Int. 24 (1), 183. 10.1186/s12935-024-03374-6 38802854 PMC11131243

[B45] Yin Z. Zhou M. Liao T. Xu J. Fan J. Deng J. (2021). Immune-related lncRNA pairs as prognostic signature and immune-landscape predictor in lung adenocarcinoma. Front. Oncol. 11, 673567. 10.3389/fonc.2021.673567 35083132 PMC8784752

[B46] Zanetti C. Krause D. S. (2020). “Caught in the net”: the extracellular matrix of the bone marrow in normal hematopoiesis and leukemia. Exp. Hematol. 89, 13–25. 10.1016/j.exphem.2020.07.010 32755619

[B47] Zangouei A. S. Zangoue M. Taghehchian N. Zangooie A. Rahimi H. R. Saburi E. (2023). Cell cycle related long non-coding RNAs as the critical regulators of breast cancer progression and metastasis. Biol. Res. 56 (1), 1. 10.1186/s40659-022-00411-4 36597150 PMC9808980

[B48] Zhang L. Zhao F. Li W. Song G. Kasim V. Wu S. (2022). The biological roles and molecular mechanisms of long non-coding RNA MEG3 in the hallmarks of cancer. Cancers (Basel) 14 (24), 6032. 10.3390/cancers14246032 36551518 PMC9775699

[B49] Zhang M. Zhang F. Wang J. Liang Q. Zhou W. Liu J. (2024). Comprehensive characterization of stemness-related lncRNAs in triple-negative breast cancer identified a novel prognostic signature related to treatment outcomes, immune landscape analysis and therapeutic guidance: a silico analysis with *in vivo* experiments. J. Transl. Med. 22 (1), 423. 10.1186/s12967-024-05237-0 38704606 PMC11070106

[B50] Zhao C. Wang Y. Tu F. Zhao S. Ye X. Liu J. (2021). A prognostic autophagy-related long non-coding RNA (ARlncRNA) signature in acute myeloid leukemia (AML). Front. Genet. 12, 681867. 10.3389/fgene.2021.681867 34276784 PMC8278057

[B51] Zhu Y. He J. Li Z. Yang W. (2023). Cuproptosis-related lncRNA signature for prognostic prediction in patients with acute myeloid leukemia. BMC Bioinforma. 24 (1), 37. 10.1186/s12859-023-05148-9 PMC9896718 36737692

